# AP2XII-1 is a negative regulator of merogony and presexual commitment in *Toxoplasma gondii*

**DOI:** 10.1128/mbio.01785-23

**Published:** 2023-09-26

**Authors:** Fuqiang Fan, Lilan Xue, Xiaoyan Yin, Nishith Gupta, Bang Shen

**Affiliations:** 1 State Key Laboratory of Agricultural Microbiology, College of Veterinary Medicine, Huazhong Agricultural University, Wuhan, Hubei, China; 2 Department of Molecular Parasitology, Institute of Biology, Humboldt University, Berlin, Germany; 3 Intracellular Parasite Education and Research Labs (iPEARL), Department of Biological Sciences, Birla Institute of Technology and Science, Pilani (BITS-P), Hyderabad, India; 4 Hubei Hongshan Laboratory, Wuhan, Hubei, China; 5 Shenzhen Institute of Nutrition and Health, Huazhong Agricultural University, Shenzhen, Guangdong, China; 6 Shenzhen Branch, Guangdong Laboratory for Lingnan Modern Agriculture, Genome Analysis Laboratory of the Ministry of Agriculture, Agricultural Genomics Institute at Shenzhen, Chinese Academy of Agricultural Sciences, Shenzhen, Guangdong, China; University of Geneva, Geneva, Switzerland

**Keywords:** AP2 transcription factors, merozoite, sexual development, MORC, merogony

## Abstract

A transition from the asexual to sexual development in the widespread intracellular pathogen *Toxoplasma gondii* requires stage switching in its feline (definitive) host, but the mechanisms governing this process remain elusive. Here, we demonstrate a novel apicomplexan-specific transcription factor, *Tg*AP2XII-1, that controls the transition of the acutely infectious tachyzoite stage to a presexual merozoite stage. The AP2XII-1 deletion mutants are elongated and replicate as merozoites by endopolygeny instead of endodyogeny, by which tachyzoites proliferate. Besides, the parasites lacking AP2XII-1 show a merozoite-type transcriptional profile. Indeed, AP2XII-1 directly targets many stage-specific genes via its interaction with the MORC complex, eventually repressing a repertoire of sex-related transcripts in tachyzoites. In conclusion, our work identifies AP2XII-1 as a merogony repressor, provides insight into the sexual commitment of *T. gondii*, and opens a gateway to culture the presexual stages of a model parasitic protist.

**IMPORTANCE:**

Sexual development is vital for the transmission, genetic hybridization, and population evolution of apicomplexan pathogens, which include several clinically relevant parasites, such as *Plasmodium, Eimeria,* and *Toxoplasma gondii*. Previous studies have demonstrated different morphological characteristics and division patterns between asexual and sexual stages of the parasites. However, the primary regulation is poorly understood. A transition from the asexual to the sexual stage is supposedly triggered/accompanied by rewiring of gene expression and controlled by transcription factors and chromatin modulators. Herein, we discovered a tachyzoite-specific transcriptional factor AP2XII-1, which represses the presexual development in the asexual tachyzoite stage of *T. gondii*. Conditional knockdown of AP2XII-1 perturbs tachyzoite proliferation by endodyogeny and drives a transition to a morphologically and transcriptionally distinct merozoite stage. The results also suggest a hierarchical transcriptional regulation of sexual development by AP2 factors and provide a path to culturing merozoites and controlling inter-host transmission of *T. gondii*.

## INTRODUCTION

*Toxoplasma gondii* is a widespread intracellular parasite of the protozoan phylum Apicomplexa, which comprises several common pathogens of animals and humans, such as *Plasmodium*, *Cryptosporidium*, *Sarcocystis,* and *Eimeria*. Infection, pathogenesis, and transmission of these parasites depend on multiple asexual and sexual stages in respective hosts. In the context of this work, the asexual tachyzoite stage of *T. gondii* can infect a broad range of intermediate hosts, including almost all warm-blood organisms. Tachyzoites can cause acute toxoplasmosis, especially in pregnant women and immunocompromised people (1 – 5). In healthy individuals, tachyzoites differentiate to form the latent tissue cysts comprising bradyzoites. *T. gondii* is also a major threat to livestock and a socioeconomic burden globally. Ingestion of livestock products infested with tissue cysts is one of the major ways of inter-host parasite transmission. Likewise, the oocysts shed by cats in the environment are an extra source of infection in other organisms. While tachyzoite-bradyzoite conversion has been studied in somewhat detail, our knowledge of transition to the presexual or sexual stages is extremely limited.

Intracellular replication of tachyzoites is marked by the emergence of two daughter parasites within a parent cell, known as endodyogeny (6). A total of 64 or more progeny are produced from one parasite in a lytic cycle lasting for about 48 h. Tight regulation of the cell cycle and budding enables tachyzoites to divide rapidly into diverse host cells of many intermediate hosts (7, 8). However, *T. gondii* has a unique definitive host, the feline, where it can initiate its sexual development in the intestine. Here, the parasite resorts to a distinct asexual expansion, termed endopolygeny, producing multiple offspring in a single division event and subsequently entering a presexual merozoite stage (9, 10). Akin to schizogony in *Plasmodium* species (11) or endopolygeny in *Sarcocystis neurona* (12, 13), merogony in *T. gondii* comprises irregular nuclear division and segregation, producing a random amount of nuclear material in a longer G1 phase, followed by budding of multiple merozoite progeny in the cytoplasm of a parent cell (6, 9, 10). However, the mechanism underlying the initiation and transformation of tachyzoites into merozoites remains unknown.

Apicomplexan parasites harbor an exclusive transcriptional factor family comprising a series of proteins with one or more plant-like AP2 domains, termed ApiAP2 transcription factors, which like their plant counterparts, could bind to specific promoter sequences and thereby regulate the expression of target genes (14). Of the 27 ApiAP2 factors known in *Plasmodium*, some are involved in initiating sexual development. For example, *Pf*AP2-G, along with other related proteins (*Pf*AP2-G2, *Pf*AP2-FG, etc.), determines gametogenesis by regulating the expression of stage-specific genes (15 – 17). Likewise, *Pf*AP2-O helps maintain the morphological stability of the ookinete stage (18). There are also several studies on ApiAP2 factors in *T. gondii*, reporting their involvement in cell cycle regulation and cysts formation. Of all 67 factors, AP2IX-9, AP2IV-4, AP2IV-3, AP2IX-4, and AP2XI-4 participate in bradyzoite and tissue cyst formation in mice by activating or repressing a number of stage-specific genes (19 – 23). Equally, AP2X-4 and AP2XII-2 control many cell cycle-dependent genes to impact the parasite growth (24, 25). Not least, AP2X-5 and AP2XI-5 can influence parasite virulence by regulating the determinants of virulence, such as ROP18 (26, 27). Nonetheless, there are many uncharacterized AP2 factors, and here, we report the role of AP2XII-1 in preventing merogony and presexual commitment/development in *T. gondii*.

## RESULTS

### AP2XII-1 is a tachyzoite-specific protein essential for parasite growth

We first analyzed the available transcriptomic data of tachyzoites, bradyzoites, and merozoites (28, 29) and found some AP2 TFs showing steep changes in abundance between these stages. Among all, AP2XII-1 was most notable because it is seemingly absent in merozoites, is barely expressed in bradyzoites, but displays a high abundance in tachyzoites (Fig. S1A), indicating its potential role in stage-specific gene expression. AP2XII-1 is a relatively large protein with 2,283 amino acid residues and contains 4 AP2 domains embedded in the center that are predicted to bind specific DNA motifs, as well as an AP2-coincident C-terminal (ACDC) domain at the tail (Fig. S1B). The ACDC domain has been reported in several AP2 factors present in apicomplexan parasites, but its function remains unknown (30).

**Fig 1:**
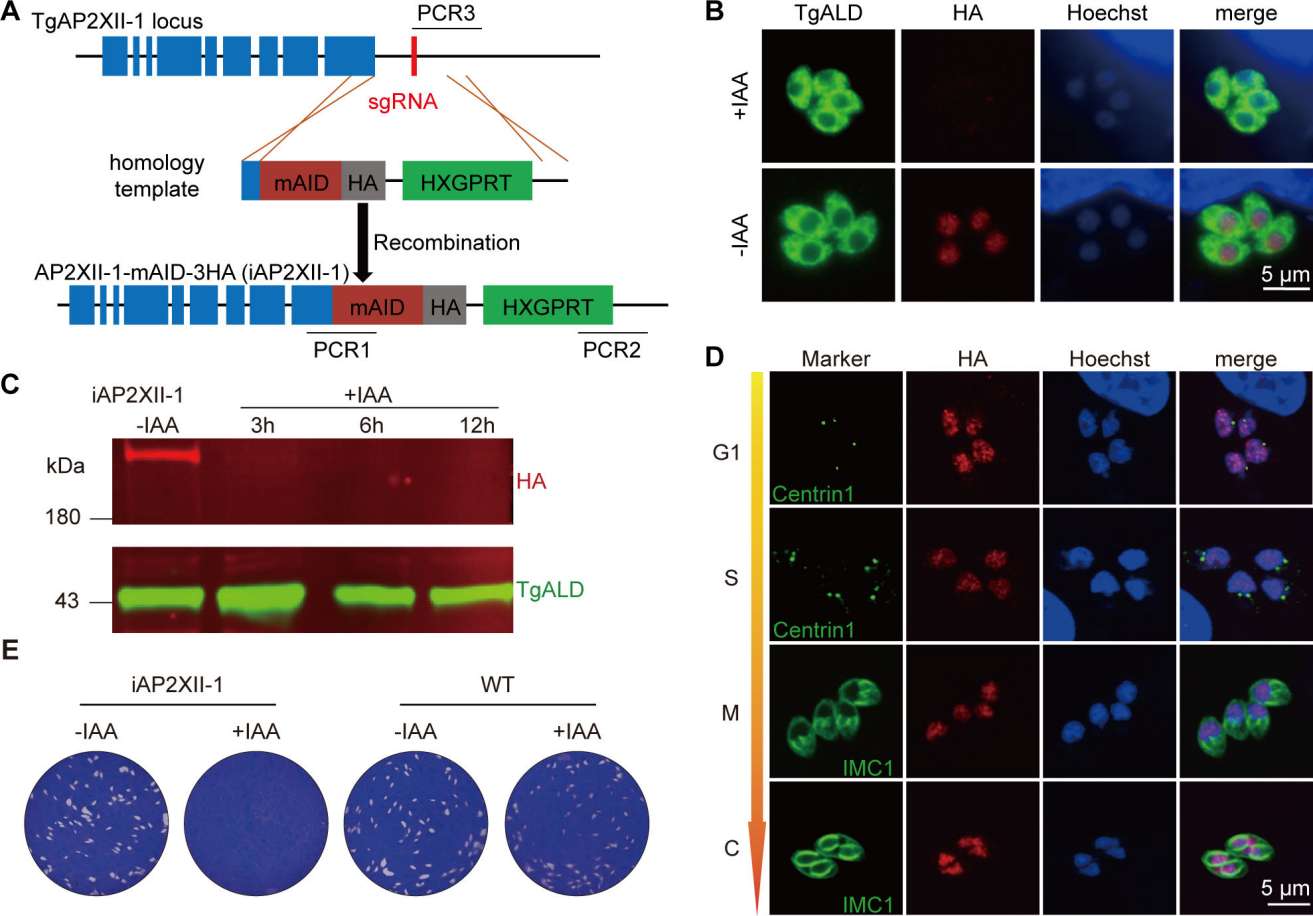
Conditional depletion of AP2XII-1 leads to growth arrest of parasites. (**A**) The strategy used to construct the conditional knockdown strain AP2XII-1-mAID-3HA (called iAP2XII-1 for short). The mAID construct (in frame with an HA tag) was fused to the C terminus of endogenous AP2XII-1 by CRISPR/Cas9-mediated homologous recombination. (**B and C**) Depletion of AP2XII-1 expression in the iAP2XII-1 strain by IAA treatment. Intracellular parasites were treated with or without 500 µM IAA and subsequently subjected to IFA (**B**) or Western blot (**C**) analyses using mouse anti-HA and rabbit anti-*Tg*ALD antibodies. (**D**) Expression of AP2XII-1 during the cell cycle of *Toxoplasma* tachyzoites, as determined by IFA examination of the iAP2XII-1 strain. *Tg*Centrin1 and *Tg*IMC1 were used to identify different stages (G1, S, M, or C stage) of the cell cycle, whereas anti-HA was used to probe AP2XII-1 expression. (**E**) Arrest of parasite growth after *Tg*AP2XII-1 depletion, as determined by plaque assays with or without IAA treatment for 7 days.

To examine the physiological roles of AP2XII-1 in tachyzoites, we engineered an auxin-regulated conditional mutant considering its low phenotypic fitness score reported in a CRISPR-based genome-wide screening (31). The transgenic parasite, RH-iKD-AP2XII-1-mAID-3HA (termed iAP2XII-1), was generated by tagging the endogenous AP2XII-1 with a mini auxin-inducible degron (mAID) and 3HA epitope at the C terminus in the RH Δ*hxgprt* TIR1 (wild-type [WT]) strain (Fig. 1A). AP2XII-1-mAID-3HA was no longer detectable by immunostaining after culturing the transgenic strain with indole-3-acetic (IAA, a type of auxin) (Fig. 1B and C), confirming a fast and efficient knockdown of the AP2XII-1 protein in iAP2XII-1. On the other hand, immunofluorescence assay showed a sustained protein expression of AP2XII-1 throughout the cell cycle in the nucleus (Fig. 1D), suggesting that its expression in tachyzoites did not fluctuate with the cell cycle. We next tested the impact of AP2XII-1 depletion on parasite growth by plaque assays (Fig. 1E). Unlike the parental strain, the iAP2XII-1 mutant did not produce any visible plaques in the presence of IAA, suggesting a vital role of AP2XII-1 during the lytic cycle of tachyzoites.

### Depletion of AP2XII-1 leads to aberrant cell division

**Fig 2:**
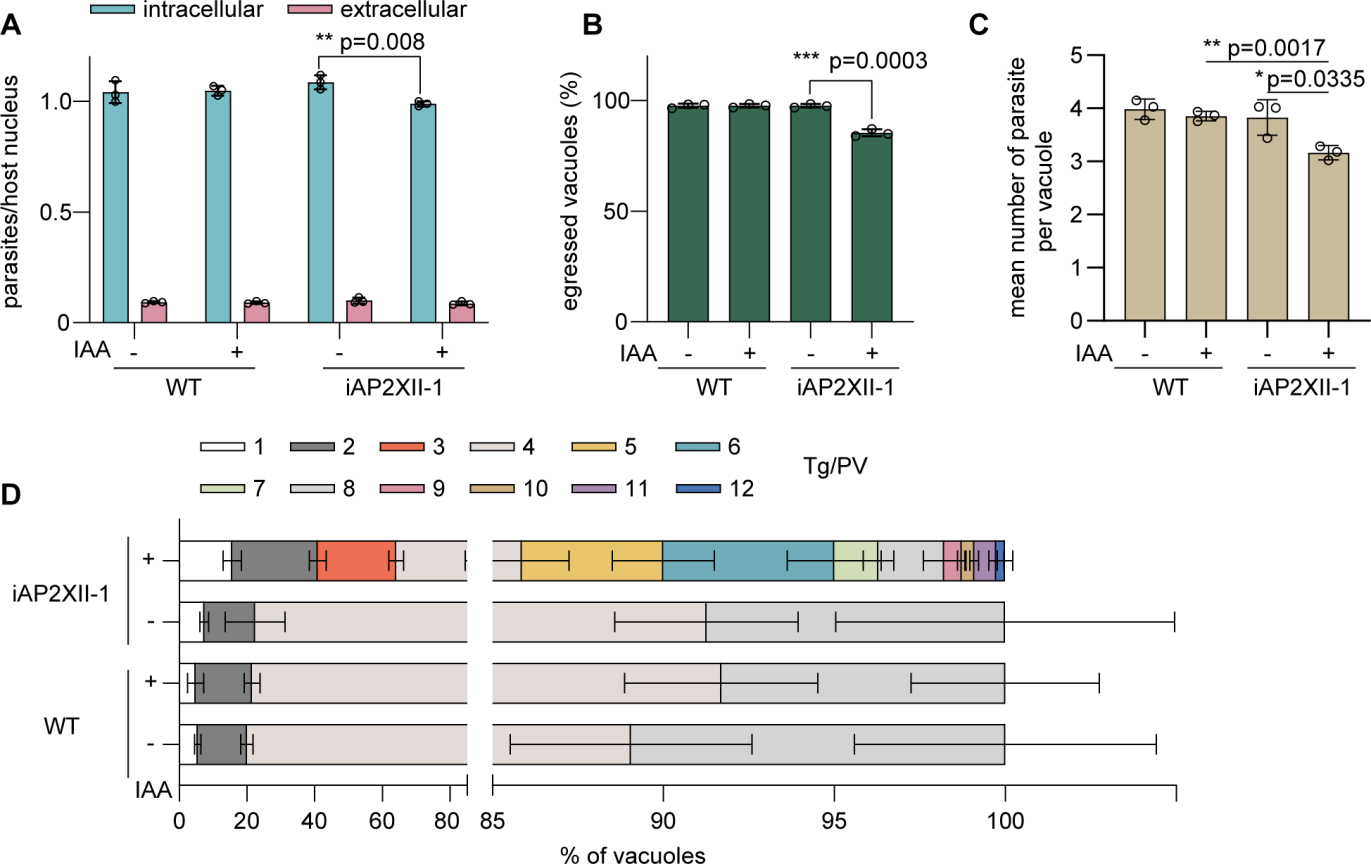
The process of endodyogeny is disrupted in parasites lacking AP2XII-1. (**A**) Invasion efficiency of indicated parasites that were pretreated with or without IAA for 42 h. Then, these pretreated strains were allowed to invade HFF cells for 20 min at 37°C, and the invasion efficiency was determined by a two-color staining assay. The number of the host cell nuclei, as well as intracellular and extracellular parasites in 30 fields, was counted for each biological replicate. Means ± SD of three independent experiments, unpaired two-tailed Student’s *t*-test. (**B**) Efficiencies of A23183-induced egress of indicated parasites that were pretreated with or without IAA for 32 h. The egress status of parasitophorous vacuoles (PVs) in 30 fields was determined for each biological replicate. Means ± SD of three independent experiments were graphed, unpaired two-tailed Student’s *t*-test. (**C**) Proliferation assays on iAP2XII-1 and RH*Δhxgprt*-Tir1 strains treated with or without IAA for 16 h (with 12 h of ±IAA pretreatment before the proliferation assay). The number of parasites in each PV was counted, and the average number of parasites in over 300 vacuoles was determined for each biological replicate. Means ± SD of three independent experiments, unpaired two-tailed Student’s *t*-test. (**D**) Replication patterns of indicated parasites with or without IAA treatment, as determined by the distribution of PVs containing different numbers of parasites after 16 h (with 12 h of ±IAA pretreatment before the proliferation assay) of intracellular growth. Means ± SEM of three independent experiments.

To gain further insights into the physiological functions of AP2XII-1, we first tested the ability of AP2XII-1-depleted parasites to invade host cells and egress out of them. The results showed that both processes were only slightly impaired in IAA-treated iAP2XII-1 parasites (Fig. 2A and B). We next examined the proliferation of the transgenic and parental strains cultured for 16 h without or with IAA. Notably, upon AP2XII-1 degradation, the mean parasite number per parasitophorous vacuole (PV) was modestly reduced (Fig. 2C). More importantly, most vacuoles of the AP2XII-1-depleted mutants harbored odd-numbered parasites (Fig. 2D), which is in sharp contrast to that of the WT strain. Normal replication of WT parasites typically increases the number of cells in PVs exponentially (multiples of 2), but this did not appear to be the case in the AP2XII-1-depleted strain. These data indicated a role of AP2XII-1 in regulating the endodyogeny, i.e., budding of daughter cells, in tachyzoites. To further investigate the unusual growth arrest, we monitored successive lytic cycles by passaging the mutant with or without IAA treatments and counting the parasite yield (Fig. S2). AP2XII-1 knockdown strain produced increasingly fewer progeny in serial passages when compared to the controls. In other words, the growth defect was not apparent until the second growth cycle after IAA treatment, even though AP2XII-1 was degraded within 3 h (Fig. 1B and C). Therefore, the growth arrest upon loss of *Tg*AP2XII-1 in tachyzoites seems to be indirect.

### AP2XII-1-knockdown mutant displays characteristics of merozoites

**Fig 3:**
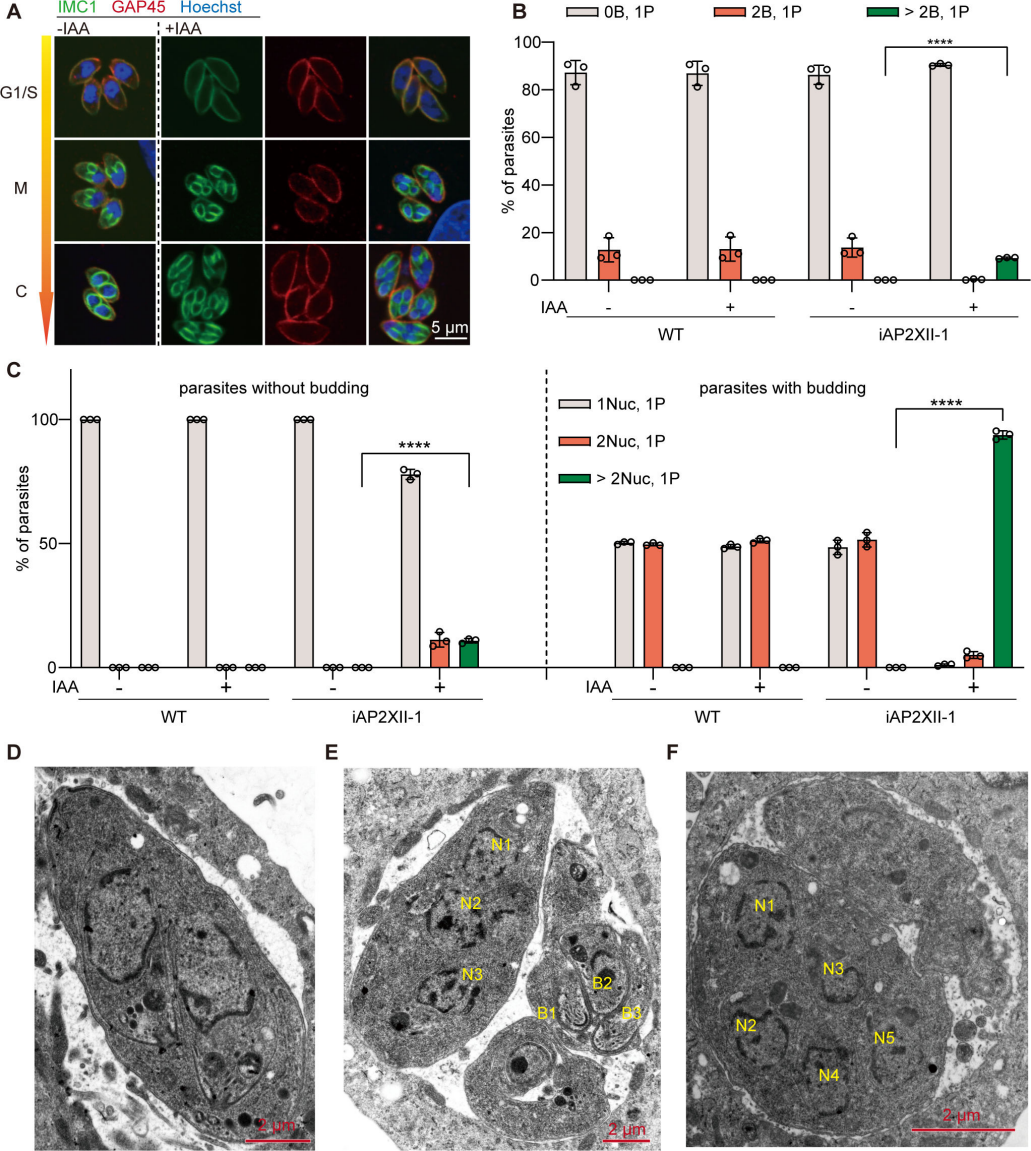
Depletion of *Tg*AP2XII-1 triggers merogony-liked division in tachyzoites. (**A**) IFA showing the patterns of daughter cell budding (as revealed by IMC1 staining) in the iAP2XII-1 strain treated with or without IAA. Note that many IAA-treated parasites contain three or more daughter cell budding inside them. (**B**) The distribution of different budding patterns in indicated strains treated with or without IAA 16 h (with 12 h of ±IAA pretreatment before assessment). 0B, 1P: no daughter cell budding in the parasite; 2B, 1P: two daughter cells budding in one parasite; >2B, 1P: three or more daughter cells budding in one parasite. More than 300 parasites were analyzed for each biological replicate. Means ± SD of three independent experiments. ****P < 0.0001, unpaired two-tailed Student’s *t*-test. (**C**) The distribution of parasites containing the indicated number of segregated nuclei, as revealed by Hoechst staining on WT or iAP2XII-1 parasites treated with or without IAA for 16 h (with 12 h of ±IAA pretreatment before assessment). 1 Nuc, 1P: one nucleus in one parasite; 2 Nuc, 1P: two nuclei in one parasite; >2 Nuc, 1P: three or more nuclei in one parasite. Parasites with or without daughter cell budding (determined by IMC1 staining) were plotted separately. More than 100 (with daughter budding) or 300 (without daughter budding) parasites were analyzed in each biological replicate. Means ± SD of three independent experiments. ****P < 0.0001, unpaired two-tailed Student’s *t*-test. (**D, E, and F**) The number of nuclei and daughter cell budding inside the iAP2XII-1 strain treated with (**E–F**) or without (**D**) IAA was determined by transmission electron microscopy. N = nucleus. B = budding daughter cells.

To explore the irregular cell division in the iAP2XII-1 strain, we performed an immunofluorescence assay using a budding-associated marker, *Tg*IMC1, and *Tg*GAP45, which are components of the inner membrane complex located underneath the plasma membrane in tachyzoites (32, 33). As expected, the iAP2XII-1 strain, in the absence of IAA, and parental parasites in the presence or absence of IAA treatments, divided by endodyogeny, where DNA replication and nuclear division were followed by cytokinesis, leading to the formation of two daughter parasites (Fig. 3A). IAA treatment of the iAP2XII-1 strain, however, resulted in multiple nuclei and buddings within a mother cell, which occurred as soon as 6 h after AP2XII-1 depletion and became very common in budding parasites 12 h after AP2XII-1 depletion (Fig S3A through C). The percentage of budding patterns revealed that almost all replicating parasites of the iAP2XII-1 strain harbored over two buds in the cytoplasm 28 h after AP2XII-1 depletion (see the “>2B, 1P” in the iAP2XII-1 + IAA group; Fig. 3B). In addition, the nuclear material accrued in the IAA-treated transgenic strain manifested as the multinucleated form in the G1/S phase of non-dividing tachyzoites (Fig. 3C), as revealed by Hoechst staining. Meanwhile, more than 90% of parasites of the AP2XII-1-depleted strain undergoing cytokinesis exhibited a multinucleated phenotype (Fig. 3C). Transmission electron microscopy confirmed the occurrence of multiple/polyploid nuclei and budding upon loss of AP2XII-1 (Fig. 3D through F).

**Fig 4:**
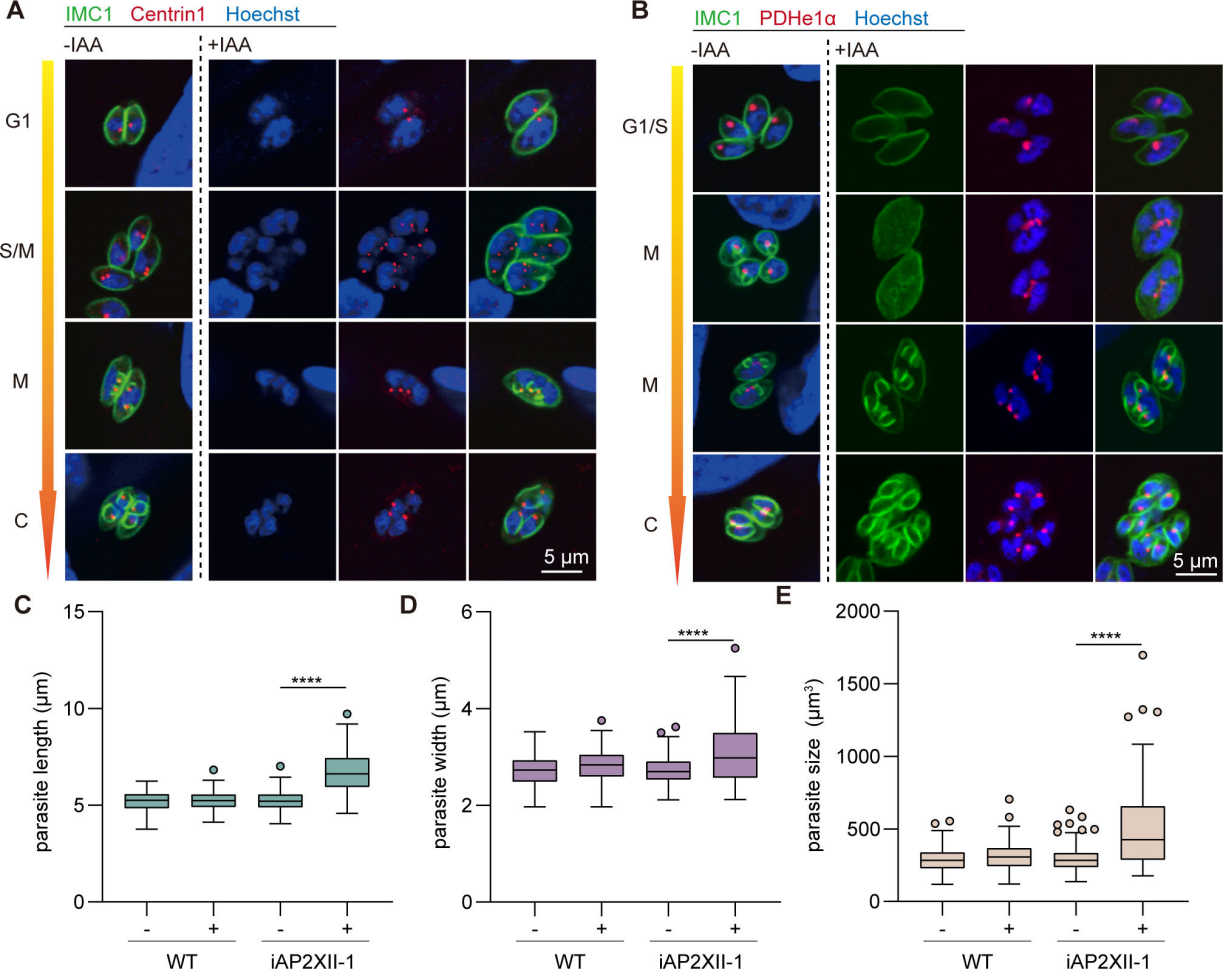
Knockdown of AP2XII-1 has no apparent effect on mitosis and apicoplast division but leads to elongated parasites. (**A and B**) Centrosome duplication and segregation, as well as apicoplast division throughout the cell cycle, were examined by staining with parasites with anti-Centrin1 (to label centrosomes) and anti-PDHe1α (to detect apicoplasts). The iAP2XII-1 strain was treated with or without IAA for 28 h before IFA analyses. (**C, D, and E**) Box plots showing the parasite length, width, and size of indicated strains treated with or without IAA treatment for 28 h. More than 50 randomly selected parasites were measured for each sample in each biological replicate. Means ± SD of three independent experiments. ****P < 0.0001, unpaired two-tailed Student’s *t*-test.

In tachyzoites, centrosome duplication in the S phase is followed by the formation of daughter cells (cytokinesis) (6). Using the centrosome outer core marker Centrin1, we monitored the pattern of cell division in the iAP2XII-1 strain (Fig. 4A). Upon exposure to IAA, multiple centrosomes could be detected in one single parasite (Fig S4A), with nuclei that were segregated or in the process of segregating. Eventually, these nuclei were successfully segregated and packaged into budding/daughter cells. These results suggest that DNA replication and subsequently mitosis were mostly normal in AP2XII-1-depleted mutants, although occasionally, we did see large and unsegregated DNA masses in non-budding parasites. On the other hand, IMC staining patterns showed that parasites underwent multiple rounds of nuclear replication and mitosis (as indicated by multiple Centrin1-stained centrosomes) before cytokinesis (Fig S4A), resulting in multiple (more than two) daughter cells budding in one mother cell (Fig. 4A). The type of mitosis and cytokinesis in the AP2XII-1-depleted mutant resembles that of schizogony seen in *Plasmodium* merozoites (11).

After centrosome segregation, organelles formed *de novo* (micronemes and rhoptries) or duplicated (apicoplasts and mitochondria) and then entered the budding cells. We followed apicoplast division in AP2XII-1-depleted parasites by staining the apicoplast-localized protein PDHe1α (34). In IAA-treated iAP2XII-1 parasites that contained multiple nuclei but where daughter cell budding not being initiated, the apicoplast was embedded in the space between nuclei (Fig. 4B). After budding was started, the apicoplast was elongated and segregated into daughter cells. Ultimately, each daughter cell acquired one apicoplast, irrespective of the number of daughter cells formed from the mother cell (Fig. 4B). As such, the number of apicoplasts in non-budding parasites was the same in AP2XII-1-expressing vs AP2XII-1-depleted strains. On the other hand, in parasites that were in the process of budding, essentially all AP2XII-1-depleted mutants contained more than two apicoplasts as subsequent endopolygeny would produce more than two daughter parasites (Fig. S4B). This phenotype is similar to the apicoplast division in merozoites of *Sarcocystis neurona* (13).

We also calculated the length and width of IMC1- and GAP45-stained parasites. We found that the iAP2XII-1 parasites treated with IAA were bigger than the same strain without IAA treatment, as well as WT parasites with or without IAA treatments (Fig. 4C and D). As such, the IAA-treated iAP2XII-1 parasites were translated into a higher volume compared to those of the control groups (Fig. 4E). All these features of AP2XII-1-depleted mutant are reminiscent of the merozoite stage of *T. gondii*, which differ from tachyzoites in cell dimensions and replicated by merogony (9, 10).

### Loss of AP2XII-1 causes the expression of merozoite-specific genes

**Fig 5:**
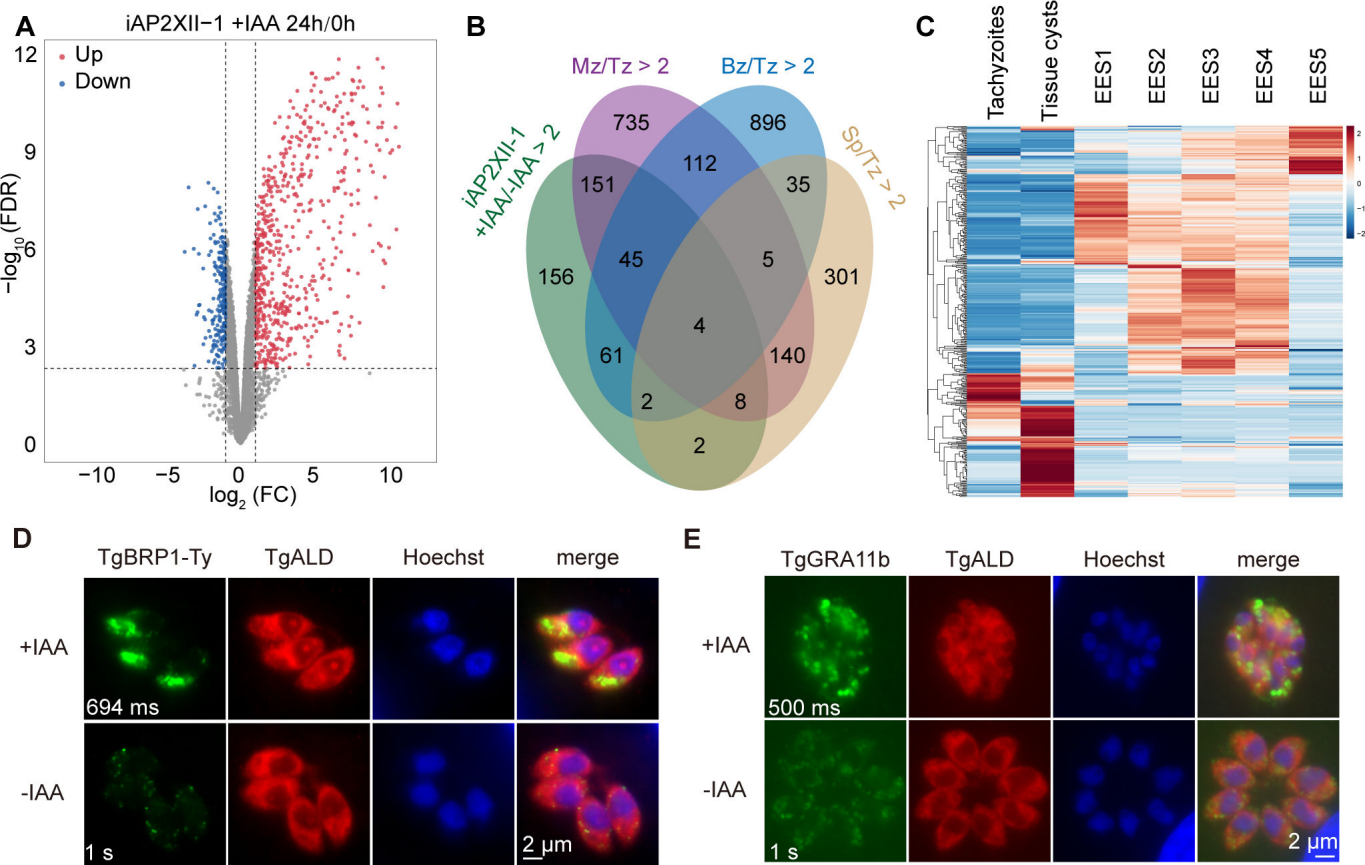
*Tg*AP2XII-1-depleted parasites exhibit a merozoite-like transcriptional profile. (**A**) Volcano plot showing the differentially expressed genes in the iAP2XII-1 strain before and after IAA treatment for 24 h, as determined by RNA-Seq. Data from three biological replicates were plotted. (**B**) Venn diagram showing the overlap of upregulated genes (>twofold) in AP2XII-1-depleted mutants with the genes that are upregulated (>twofold) in wild-type merozoites (Mz), bradyzoites (Bz), or sporozoites (Sp) compared with wild-type tachyzoites; data for which are from published data sets available in ToxoDB (28, 35, 36). (**C**) Life cycle expression patterns of the 399 genes that were upregulated in AP2XII-1-depleted mutants were plotted as a heatmap using data from ToxoDB (29). (**D and E**) IFA that shows the protein expression of *Tg*BRP1 and *Tg*GRA11b in the iAP2XII-1 strain treated with or without IAA for 24 h. GRA11b was directly detected by an anti-GRA11b antibody, whereas BRP1 was detected by a Ty antibody in an iAP2XII-1/*Tg*BRP1 Ty transgenic strain. The exposure times for acquiring images from the green channel were indicated on the lower left of the images.

To check the impact of AP2XII-1 depletion on gene expression in *T. gondii*, total RNA of the iAP2XII-1 strain treated with IAA for different periods (0 h, 12 h, and 24 h) was extracted and subjected to RNA-Seq analyses to compare the messenger RNA levels for each gene. We found that transcriptional profiles changed gradually with the duration of IAA exposure. The significantly altered transcripts were identified by a combination of Salmon and edgeR suites (FDR ≤0.005; fold change ≥2), resulting in 429 and 184 genes whose abundance was increased and decreased, respectively, upon depletion of AP2XII-1 by IAA treatment for 24 h (Fig. 5A; Table S2). Interestingly, when the genes with increased RNA abundance after AP2XII-1 depletion were compared to those of the published data on gene expression differences between wild-type tachyzoites and merozoites (28), sporulated oocysts and tachyzoites (35), and tissue cysts and tachyzoites (36), we observed that 63% (271 of 429) of them matched the upregulated genes during the pre-sexual development or chronic infection stages, and the majority (208 of 271) were upregulated in merozoites (Fig. 5B). A heatmap comparing the expression patterns of the 378 genes with increased RNA abundance in the AP2XII-1-depleted mutants during the parasite’s life cycle revealed that the majority of them were upregulated in early entero-epithelial stages (EES1-5), and a small fraction was upregulated in bradyzoites (Fig. 5C). These results suggest that AP2XII-1 suppressed the expression of merozoite- and bradyzoite-specific genes in tachyzoites.

To further analyze the 429 genes with increased RNA abundance in the AP2XII-1-depleted mutant, they were categorized into different groups based on their sequence features. More than half (234) of these genes are hypothetical proteins with unknown functions. For the rest of the 195 genes, significant enrichments were found in clusters containing SAG-related sequence proteins (SRS), *Toxoplasma* family A-E proteins, micronemal proteins (MIC), dense granule proteins (GRA), and AP2 factors (Fig. S5), many of which are merozoite-specific genes. To test the upregulation of these genes at the protein level, we selected two genes, *TgBRP1* and *TgGRA11b*, both of which are abundantly expressed in wild-type merozoites and were highly increased at the mRNA level in AP2XII-1-depleted mutants. The *TgBRP1* gene was tagged with a Ty epitope at the C terminus of its endogenous gene locus in the iAP2XII-1 strain. Subsequently its expression was assessed by IFA after treating the parasites with or without IAA. The results showed that *Tg*BRP1-Ty could be easily detected in AP2XII-1-depleted parasites but not in parasites expressing AP2XII-1 (Fig. 5D). To estimate the protein level of GRA11b, a GRA11b-specific antibody was used to probe its expression in the iAP2XII-1 strain with or without IAA treatment. Again, its expression was significantly increased after treating the parasites with IAA (Fig. 5E). To further analyze the protein-level changes of GRA11b after AP2XII-1 depletion at the bradyzoite stage, the ME49 iAP2XII-1 strain (described below) was first cultured in alkaline medium for 3 days and then treated with or without IAA for an additional 48 h. Subsequently, the protein levels of GRA11b were probed by IFA as the above. The results showed that the GRA11b level was also increased in AP2XII-1-depleted mutants at the bradyzoite stage (Fig. S6). Together, these results suggest that expressions of BRP1 and GRA11b were highly induced upon depletion of AP2XII-1, at both transcriptional and translational levels.

In contrast to the genes with increased mRNA levels, the ones with decreased mRNA abundance in AP2XII-1-depleted mutants exhibited evident bias in tachyzoites, meaning that their expression was higher in tachyzoites than in merozoites in WT parasites (Fig. S7A). Interestingly, the expression of most of the downregulated genes oscillated in a cell cycle-dependent manner in WT tachyzoites (37) (Fig. S7B). Among these, AP2IX-5 is known to be crucial for the parasite division, and AP2XII-2 is reported to regulate the expression of merozoite-specific genes (38, 39). Quantitative RT-PCR analyses of selected downregulated genes confirmed decreased transcript levels of AP2IX-5, AP2XII-2, IMC9, MIC10, RON5, ROP18, ROP19A, and ROP34 after knockdown of AP2XII-1 (Fig. S7C), which is also consistent with their downregulation in merozoites derived from cat intestines (28).

### AP2XII-1 binds to the promoter regions of target genes and interacts with other gene regulation factors to regulate the expression of merozoite specific genes

**Fig 6:**
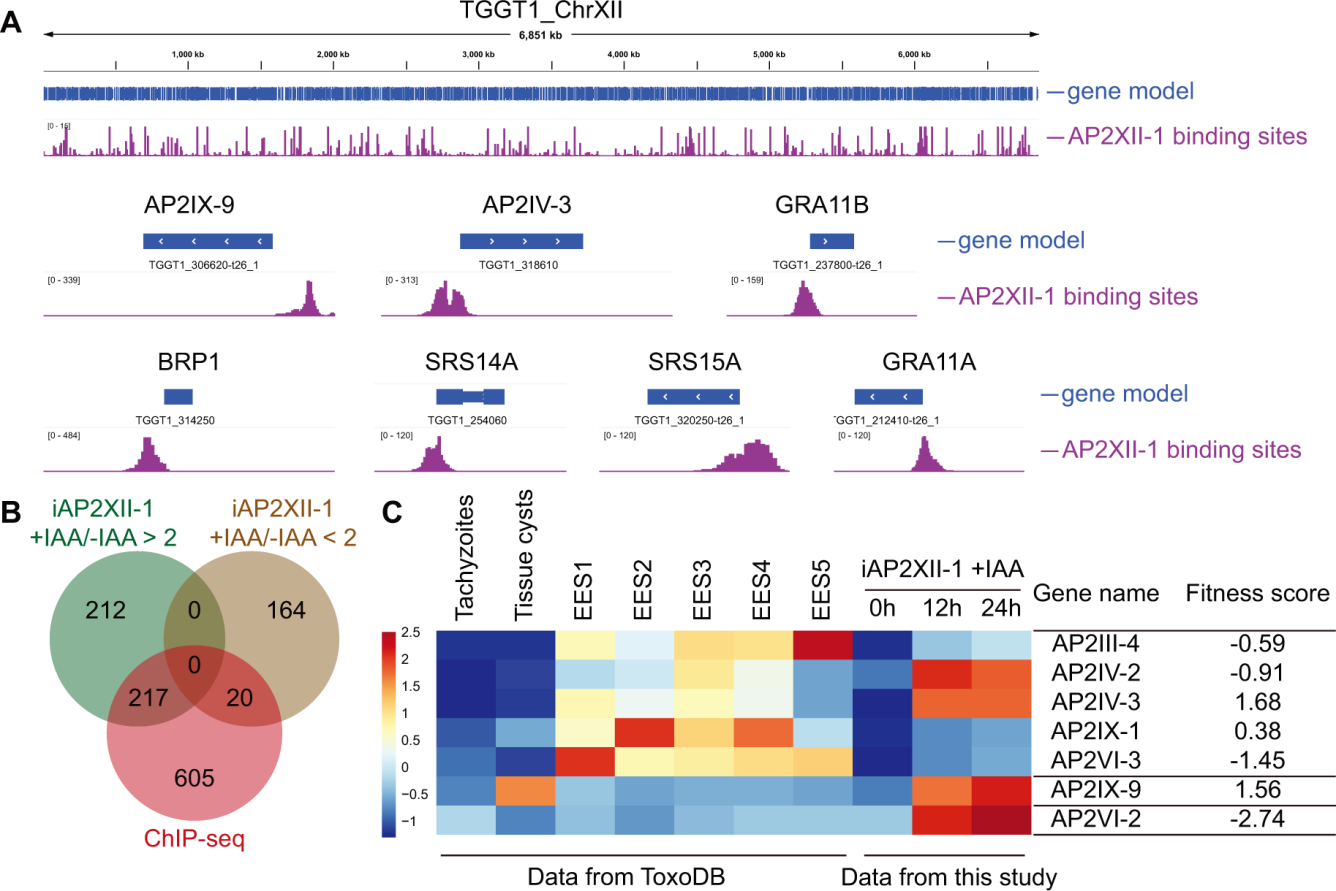
AP2XII-1 binds to promoter regions of target genes to regulate their expression. (**A**) Genomic sites bound by *Tg*AP2XII-1 revealed by ChIP-Seq using the iAP2XII-1 strain. The bind sites (peaks) on chromosome XII were visualized in the IGV browser and used as an example for demonstration. (**B**) Venn diagram showing the overlap of genes upregulated or downregulated (determined by RNA-Seq) in the AP2XII-1 depletion mutant and those that were directly targeted by AP2XII-1 (determined by ChIP-Seq). (**C**) Transcriptional patterns of the seven AP2 factors that were directly targeted by AP2XII-1 during the life cycle of *T. gondii* and in the iAP2XII-1 strain treated with or without IAA.

Transcriptomic analyses revealed the gene expression changes after AP2XII-1 depletion. To see which of these differentially expressed genes are directly targeted by AP2XII-1, we used chromatin immunoprecipitation, followed by deep sequencing (ChIP-Seq) to assess the binding positions of AP2XII-1 in the parasite’s genome (Fig. 6A). The DNA bound to AP2XII-1 was co-precipitated by an HA antibody in the iAP2XII-1 strain and then identified by sequencing. The results showed that AP2XII-1 was significantly enriched in promoter regions of 842 genes (Fig. 6A and B; Table S3). More importantly, 50.5% upregulated (217 of 429) and 10.8% downregulated (20 of 184) genes in the AP2XII-1 depletion mutant were directly targeted by AP2XII-1 (Fig. 6B), including merozoite- and/or bradyzoite-specific genes like GRA11a, BRP1, GRA11b, SRS14A, and SRS15A, as well as AP2 factors that are known to have roles in regulating parasite development, such as AP2IX-9 and AP2IV-3 (Fig. 6A). These results indicated direct roles of AP2XII-1 in stage-specific gene expression. In addition, of the 12 AP2 factors that were upregulated in the AP2XII-1 mutant, seven of them were directly targeted by AP2XII-1 (Fig. 6C). Significantly, five out of the seven AP2XII-1-regulated AP2 factors exhibited higher expression in merozoites than other stages, and they probably had limited contributions to tachyzoite growth, as judged by their genome-wide phenotypic scores (Fig. 6C). On the other hand, AP2IX-9 accumulated in bradyzoites, and it has been shown to be critical for tissue cyst development (19).

To further check the possible mechanisms by which AP2XII-1 used to regulate target gene expression, we pulled down AP2XII-1 from the iAP2XII-1 strain by immunoprecipitation and identified its potential binding partners by mass spectrometry. Compared with the control group that used mouse IgG for immunoprecipitation, the experimental group (which used anti-HA for immunoprecipitation) contained several proteins that were not identified in the control. These includes AP2XII-1 itself, as well as a hypothetical protein (TGGT1_209500), *Tg*MORC (a member of the transcriptional repressor complex [40]) and an AP2 transcriptional factor (*Tg*AP2XI-2) (Table 1). Given the known role of *Tg*MORC in regulating the expression of merozoite-specific genes (40), these data suggest that AP2XII-1 forms a complex with other transcriptional and epigenetic factors to control the development of pre-sexual stages.

**TABLE 1:** Selected proteins co-precipitated with AP2XII-1

Gene ID	Protein	# of unique peptides^*a*^
TGGT1_218960	AP2XII-1	19
TGGT1_209500	Hypothetical protein	11
TGGT1_305340	MORC	8
TGGT1_310900	AP2XI-2	6

^*a*^ Number of unique peptides derived from co-IP and MS of the experimental group (use anti-HA for immunoprecipitation). For all hits listed here, no unique peptides were found in the control group (use mouse IgG for immunoprecipitation).

### Depletion of AP2XII-1 causes spontaneous bradyzoite transition in a type II strain

**Fig 7:**
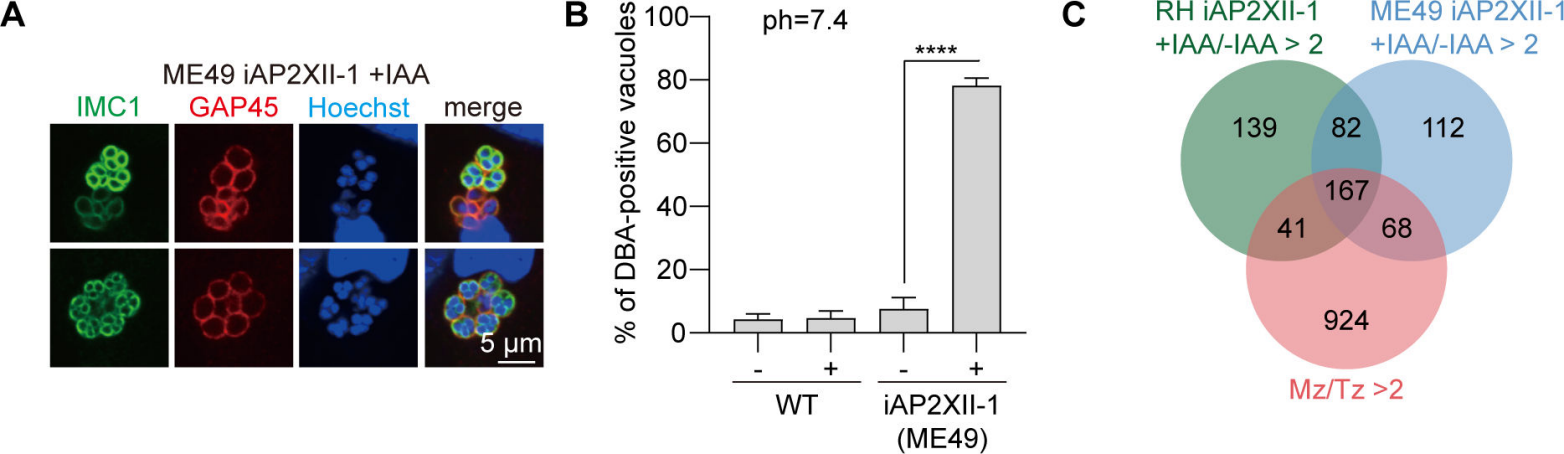
Effect of *Tg*AP2XII-1 ablation on the division and bradyzoite development of the type II strain ME49. (**A**) IFA showing a merogony-like proliferation phenotype in the ME49-iAP2XII-1 strain treated with IAA, as determined in Fig. 3A. (**B**) The percentage of DBA-positive PVs in indicated strains treated with or without IAA. Parasites were cultured under standard tachyzoite growth conditions. Means ± SD of three independent experiments. ****P < 0.0001, unpaired two-tailed Student’s *t*-test. (**C**) Venn diagram showing the overlap of genes that are upregulated (>twofold) in wild merozoites (28) and IAA-treated RH-iAP2XII-1 or ME49-iAP2XII-1.

To further decipher the developmental role of AP2XII-1, an auxin-inducible degron system was deployed in the type II strain ME49, which is able to complete the full life cycle, unlike the RH strain used above that can only grow as tachyzoites. Similar to the AP2XII-1 depletion mutant generated in RH (described above in Fig. 3 and Fig. 4), the ME49/iAP2XII-1 strain is divided by merogony and formed elongated merozoites upon depletion of AP2XII-1 in the presence of IAA (Fig. 7A). In both type I and type II strains, we observed that the parasites in almost all parasitophorous vacuoles displayed a disordered arrangement akin to encysted bradyzoites after depletion of AP2XII-1. We, therefore, tested whether the parasites lacking AP2XII-1 show the features of bradyzoites by staining them with FITC-conjugated DBA, which reacts with the wall of cysts. As expected, we found that over 90% of ME49/iAP2XII-1 vacuoles exhibited positive DBA staining after 40 h of treatment with IAA, which was in sharp contrast to only 3%–5% DBA-stained vacuoles in untreated transgenic or control parental strains (Fig. 7B). These results demonstrated a very high rate of spontaneous bradyzoite transition in AP2XII-1-depleted mutants. RNA-Seq was also performed to detect the gene expression changes in the ME49 mutant after AP2XII-1 degradation. Consistent with AP2XII-1 depletion in the type I strain RH, ME49/iAP2XII-1 treated with IAA also increased the expression of many merozoite-specific genes at the tachyzoite stage (Fig. 7C). These data further confirmed the role of *Tg*AP2XII-1 regulating pre-sexual development of *T. gondii*.

## DISCUSSION

The natural life cycle of *T. gondii* shuttles between the definitive and intermediate hosts and involves the formation of multiple infectious stages, including tachyzoites, bradyzoites, and merozoites. While the tachyzoite and bradyzoite stages have been relatively better studied due to the ease of *in vitro* propagation, the merozoite stage preceding the sexual development in the feline intestine remains enigmatic because of animal ethics and non-permissive culture. These stages could be distinguished by various characteristics. Here, we show that loss of *Tg*AP2XII-1 drives tachyzoites to divide by endopolygeny and form multinucleated parasites resembling merogony in merozoites. In addition, *Tg*AP2XII-1 directly and indirectly regulates the expression of hundreds of merozoite-specific genes and its depletion causes increased expression of such genes, suggesting that *Tg*AP2XII-1 is a negative regulator of merogony and sexual commitment in *T. gondii*. On the other hand, although parasites lacking AP2XII-1 develop to a merozoite-like stage, they eventually perish, perhaps due to a deficiency in the culture condition that did not support parasite viability after prolonged merogony. It has been suggested that the nutritional environment of the cat intestine, such as high levels of linoleic acid, is critical for merozoite growth and sexual development of *T. gondii* (41). Nonetheless, this study paves the way to explore the pre-sexual developmental mechanism and merozoite biology without a need for a feline host.

**Fig 8:**
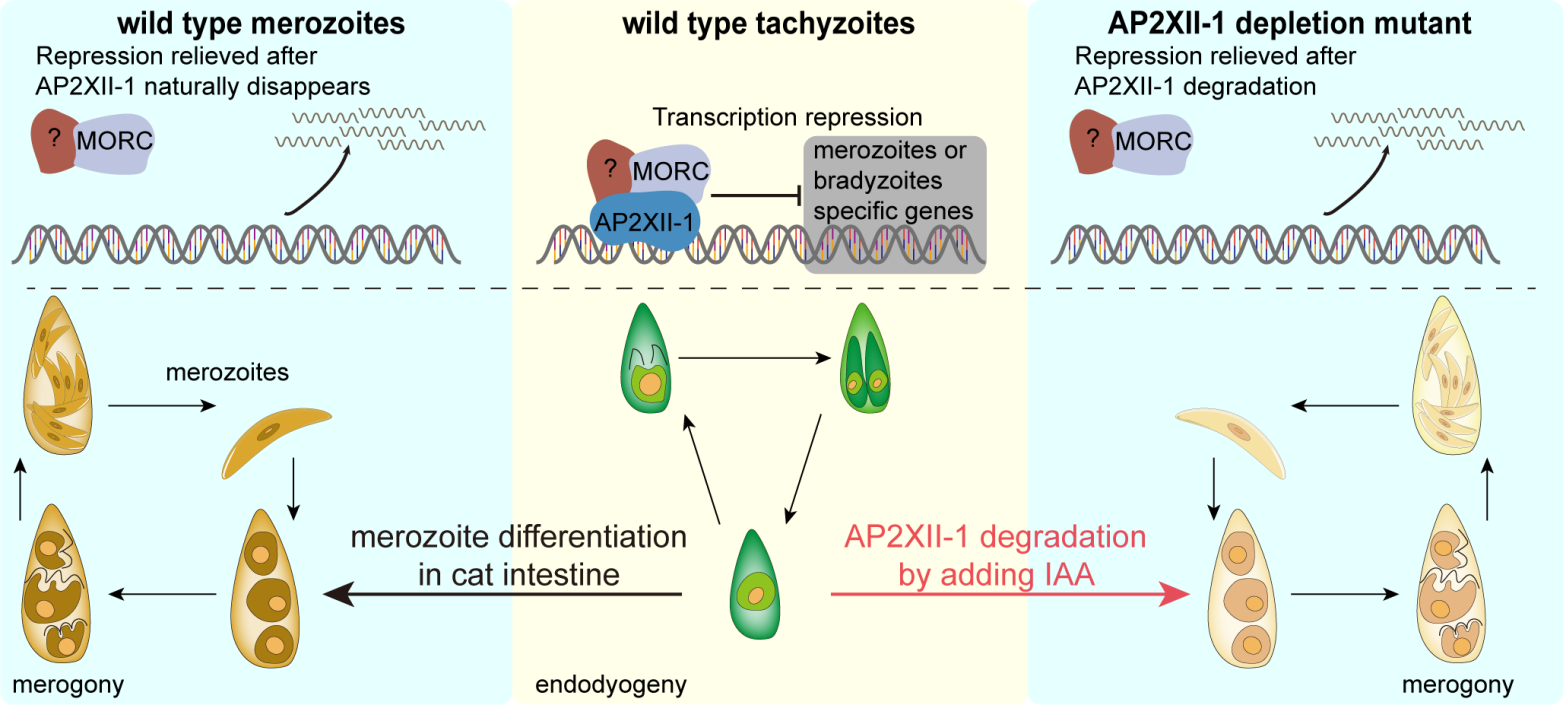
Model for the regulation of merogony and pre-sexual commitment by AP2XII-1. At the tachyzoite stage, AP2XII-1 forms a complex with MORC and other gene regulation factors (such as AP2 factors, denoted by the ? symbol) to suppress the expression of merozoite-specific genes and to prevent parasite proliferation by merogony. In the AP2XII-1-depleted mutant, parasites displayed a merozoite-like replication and gene expression profile, as the inhibitory effect of AP2XII-1 is relieved. In wild-type merozoites, the expression of AP2XII-1 is shut down by unknown mechanisms. As such, its inhibition on merogony and merozoite-specific gene expression are removed and merozoite development proceeded.

The conversion of tachyzoites or bradyzoites to merozoites requires activation of merogony-specific genes that we report here be controlled by the AP2XII-1 transcription factor. A rewiring of the transcriptome in AP2XII-1-depleted parasites resembles the gene expression in merozoites in a temporal manner. For example, the levels of a number of SRS proteins and secretory proteins (MIC17A, GRA11a, and GRA11b) increased dramatically upon loss of AP2XII-1. Such a transcriptional profile, along with morphological features of the mutant, strongly suggests a crucial role of AP2XII-1 in inhibiting the development of mature merozoites under tachyzoite growth conditions. In naturally occurring merozoites, the expression of AP2XII-1 is extremely low; thus, its inhibitory effect on merozoite differentiation is relieved and sexual development can proceed (Fig. 8). AP2XII-1 probably works together with other factors to inhibit merozoite development under normal conditions. First, AP2XII-1 appears to interact with MORC, which has been shown to interact with and recruit histone deacetylase 3 (HDAC3) to suppress the expression of many developmentally regulated genes, including a large set of merozoite-specific genes (40). Depletion of AP2XII-1 caused a similar gene expression change as the knockdown of MORC (40). Thus, it is possible that through interaction with MORC, AP2XII-1 brings MORC to the target sites to inhibit their expression. Second, AP2XII-1 also directly regulates the expression of a number of AP2 factors (Fig. 6C), including five merozoite-specific AP2 factors and the bradyzoite-specific AP2IX-9 that has been well demonstrated to affect bradyzoite development (19). As such, AP2XII-1 may be positioned at an upstream point of transcriptional cascades that silence merogony in tachyzoites.

In conclusion, this study reveals that AP2XII-1 suppresses merozoite differentiation and pre-sexual commitment in tachyzoites by repressing merozoite-specific and possibly by inducing tachyzoite-specific genes. It interacts with the MORC epigenetic repressor complex and other factors to regulate the synthesis of many typical proteins needed for merozoite formation. Besides, AP2XII-1 controls the expression of a slew of AP2 factors that are either inoperable or absent in tachyzoites but become functional in merozoites. Its own expression is tightly regulated by a yet-unknown mechanism to be restricted to the tachyzoite stage. Therefore, its inhibitory effect on merogony and merozoite development is the strongest at the tachyzoite stage and is relieved in merozoites. In a nutshell, our work provides a foundational framework for prospective studies on the regulation of the sexual development in *Toxoplasma gondii* and offers a gateway to achieve *in vitro* propagation of merozoites and target the inter-host transmission of this clinically relevant parasitic protist.

## MATERIALS AND METHODS

### Parasites and culture conditions

Parental strains include RH Δ*hxgprt* TIR1 and ME49 TIR1, which both were constructed by introducing the pTUB1:OsTIR1-3FLAG-CAT fragment (amplified from Addgene plasmid no. 87258) into RH Δ*hxgprt* or ME49 strains as described previously (42). Other transgenic strains were constructed as described below.

All *Toxoplasma* tachyzoites used in this study were maintained in human foreskin fibroblasts (HFF) (ATCC, USA) cultured at 37°C with 5% CO_2_ in complete Dulbecco’s modified Eagle medium (DMEM) (Sigma-Aldrich, USA) supplemented with 10% heat-inactivated fetal bovine serum (FBS) (Gibco, USA), 100 unit/mL penicillin, and 100 µg/mL streptomycin. Prior to the infection with confluent HFF monolayers, parasites were scraped and syringe lysed using a 22-gauge needle, filtered by filtration through 3 µm polycarbonate membranes.

### Construction of plasmids and transgenic strains

The locus-specific CRISPR plasmids were generated according to previously described protocols (43). Other plasmids were constructed by multi-fragment cloning using the ClonExpress MultiS Cloning Kit (Vazyme Biotech, China). Oligonucleotides listed in Table S1 were synthesized by Tsingke Biotechnology Limited (Beijing, China). The pUC19-RHAP2XII-1-mAID-HXGPRT was constructed by recombining the fragments of mAID-3HA-HXGPRT, 5′ and 3′ homologous arms of AP2XII-1 into the plasmid pUC19. The mAID construct was amplified from pTUB1::YFP-mAID-3HA-HXGPRT (Addgene plasmid no. 87259), and the 5′ and 3′ homologous arms were amplified from genomic DNA of RH Δ*hxgprt* TIR1. The pUC19-ME49AP2XII-1-mAID-HXGPRT plasmid was constructed using the same approach as pUC19-RHAP2XII-1-mAID-HXGPRT. The only change was that the 5′ and 3′ homologous arms were amplified from ME49 TIR1 instead of RH. For the construction of BRP1-Ty in the RH iAP2XII-1 strain, the pUC19-BRP1-Ty-DHFR* was also constructed using multi-fragment cloning. The fragment Ty-DHFR* was amplified from pUC19-Ty-DHFR*, and the two homologous arms were amplified from the genomic DNA of the RH iAP2XII-1 strain.

All transgenic strains were constructed by CRISPR/Cas9-mediated site-specific gene editing (44). The homologous recombination fragments were amplified from the homologous template plasmids constructed as above and co-electroporate with corresponding CRISPR plasmids into appropriate parental strains. The transfectants were selected by suitable drugs according to the drug-resistance genes in the homologous fragments. An amount of 25 µg/mL mycophenolic acid (MPA) and 50 µg/mL xanthine (Sigma-Aldrich, USA) were used for the HXGPRT gene, and 1 mM pyrimethamine (Sigma-Aldrich, USA) was used for the DHFR* gene. Single clones were segregated by limiting dilution and screened by diagnostic PCRs.

### Immunofluorescent assays and Western blots

IFAs and Western blots that examined protein expression were performed according to established protocols (43, 45). The primary antibodies used in this study include mouse anti-Ty (the BB2 clone of anti-Ty1, provided by Dr. David Sibley, Washington University in St. Louis, USA), rabbit anti-*Tg*ALD polyclonal antibody, rabbit anti-*Tg*IMC1 polyclonal antibody (provided by Dr. Qun Liu from China Agricultural University, China), mouse anti-HA (the TANA2 clone of anti-HA, MBL International Corporation, Japan), mouse anti-*Tg*GAP45 serum (32), mouse anti-*Tg*PDHe1α serum, and mouse anti-Centrin1 monoclonal antibody (the 20H5 clone, Sigma-Aldrich). The secondary antibodies used in this study include IRDye 680RD goat anti-rabbit IgG, IRDye 800CW goat anti-mouse IgG (LI-COR Biosciences, USA), HRP-labeled goat anti-rabbit IgG, HRP-labeled goat anti-mouse IgG (Beyotime Biotechnology, China), Alexa Fluor 488-conjugated goat anti-mouse IgG, Alexa Fluor 594-conjugated goat anti-mouse IgG, Alexa Fluor 488-conjugated goat anti-rabbit IgG, and Alexa Fluor 594-conjugated goat anti-rabbit IgG (Fisher Scientific, USA). IFA images were acquired from the Olympus FluoView FV1000 Confocal Microscope (Olympus Life Science, Japan) or Olympus BX53 Microscope (Olympus Life Science, Japan) equipped with an AxioCam 503 mono camera (Zeiss, Germany). Western blots were scanned by an Amersham Typhoon 5 imager (GE Healthcare, UK).

### Parasite growth and fitness assessment

Plaque assays were used to assess the overall growth of parasites. Intracellular replication assays were used to determine the efficiency of parasite proliferation after successful invasion. Two-color invasion assays and induced egress assays that estimated efficiencies of parasite invasion into and egress out of HFF cells were performed as previously described (25, 45, 46). To assess parasite propagation after prolonged depletion of *Tg*AP2XII-1, 1.2 × 10^6^ of tachyzoites of the RH iAP2XII-1 or RH Δ*hxgprt* TIR1 strains were allowed to infect confluent HFF monolayers seeded in T25 flasks and cultured for 48 h with or without IAA treatment. Then, the parasites were harvested and counted using a hemocytometer. Meanwhile, 1.2 × 10^6^ parasites were passed into fresh T25 flasks and cultured for another 48 h using the same growth condition as in the first 48 h. Subsequently, the parasites were harvested and counted.

To check the size of *T. gondii* parasites, indicated strains were treated with or without IAA for 40 h, fixed by 4% paraformaldehyde, stained by mouse anti-*Tg*GAP45 and rabbit anti-*Tg*IMC1, and then imaged by an Olympus BX53 Microscope equipped with an AxioCam 503 mono camera. Then, the parasite length and width were measured by the ImageJ software. The parasite size was calculated as follows: parasite size = 4/3 × π × 1/2 parasite length × 1/2 parasite width × 1/2 parasite width.

### Transmission electron microscope

To prepare samples for the transmission electron microscope, RH iAP2XII-1 was allowed to invade fresh confluent HFF monolayers in T75 flasks and grown with or without IAA for 24 h. Then, all samples were scraped and centrifuged at 1,000 *g* for 5 min before fixation with 0.25% (wt/vol) glutaraldehyde in 0.1 mol/L PBS at 4°C overnight. Subsequently, all samples were washed with PBS, fixed in 1% (wt/vol) OsO_4_, and processed for sectioning as previously described (47). The samples were sectioned into ultrathin sections (60–70 nm thick) using a diamond knife on a UC6 Ultratome (Leica, Germany) and stained with 2% (vol/vol) uranyl acetate. The images were viewed and collected with a Hitachi transmission electron microscope (TEM; H-7650; Hitachi, Japan) at 80 kV.

### RNA-seq and qRT-PCR

To prepare samples for RNA-seq analyses, total RNAs were extracted from freshly egressed parasites using TRIzol Reagent according to the manufacturer’s instructions (TransGen Biotech, China). DNA digestion by DNaseI was carried out after RNA extraction. RNA quality was determined by a Nanodrop OneC spectrophotometer (Thermo Fisher Scientific, USA). RNA Integrity was confirmed by gel electrophoresis in a 1.5% agarose. RNAs were finally quantified with the Qubit RNA Broad Range Assay Kit (Life Technologies, USA) and the Qubit3.0 Fluorometer (Invitrogen, USA).

For RNA-seq, 2 μg total RNA of each sample was used for RNA sequencing library preparation using the KCTM Stranded mRNA Library Prep Kit (Wuhan Seqhealth Co. Ltd. China) for Illumina sequencing, following the manufacturer’s instructions. PCR products with the sizes of 200–500 bps were enriched, quantified, and sequenced on the DNBSEQ-T7 sequencer (MGI Tech Co. Ltd., China) with the PE150 model. Three independent replicates were performed. Raw data were trimmed and quality controlled by SeqPrep and Sickle with default parameters. Then, clean data were mapped to reference genome GT1 (ToxoDB) using Salmon (version 1.9) (48). The transcript per million reads (TPM) method was used to calculate the transcript levels of each gene. Differentially expressed gene (DEG) analysis was performed by edgeR with a false discovery rate (FDR) cutoff of 0.005 and a minimum fold change of 2 (https://bioconductor.org/packages/release/bioc/html/edgeR.html). Raw data of RNA-Seq obtained in this study have been deposited to the GEO database under the accession number GSE224934.

For quantitative RT-PCR that compared the transcript levels in wild-type and AP2XII-1 depletion mutants, RNA samples extracted as above were analyzed by the Fast SYBR method (Vazyme Biotech Co., China) performed in the QuantStudio 3 Real-Time PCR System. All primers are listed in Table S1.

### Chromatin immunoprecipitation and data analyses

To perform chromatin immunoprecipitation, intracellular parasites of RH iAP2XII-1 were fixed with 1% formaldehyde for 10 min. A final concentration of 0.125 M glycine was added to quench the fixation. Then, all samples were scraped and syringe lysed using a 22-gauge needle, filtered by filtration through 3 µm polycarbonate membranes. The purified parasites were lysed using the ChIP lysis buffer (50 mM HEPES, 150 mM NaCl, 1% NP40, 0.1% SDS, 0.1% sodium deoxycholate, 1 mM EDTA, pH 8, and Roche protease inhibitor cocktail) for 30 min, and the lysates were sonicated using the bioruptor UCD-200 for 16 min at 4°C with a 30-s on/off cycle. For each immunoprecipitation, about 30 µg chromatin extracts were mixed with 3 µg mouse anti-HA antibody (MBL International Corporation, Japan) and incubated overnight at 4°C with rotation. Then, protein A-conjugated dynabeads (Thermo Fisher, USA) were added to capture chromatin antibody complexes for 4 h at 4°C. The precipitated DNA was subjected to high-throughput DNA sequencing analyses. The libraries for sequencing were constructed using the VAHTS Universal DNA Library Prep Kit for Illumina V3 (Vazyme, China). Fragments with the sizes of 200–500 bps from the libraries were enriched, quantified, and sequenced on the DNBSEQ-T7 sequencer (MGI Tech Co. Ltd., China) with the PE150 model. To analyze the ChIP-Seq results, raw sequencing data were filtered by Trimmomatic (version 0.36) (49), and reads contaminated with adaptor sequences were trimmed. The clean reads were aligned to reference genome GT1 (ToxoDB) using the STAR software (version 2.5.3a) (50) with default parameters. The MACS2 software (version 2.1.1) (51) was used for peak calling. Peaks enriched in both ChIP-Seq experiments were considered binding sites of AP2XII-1. Then, the Excel or bigwig files containing the enriched peaks were generated, and the bigwig files were used to visualize peak abundance in the Integrative Genomics Viewer (IGV) as loaded tracks. The original ChIP-Seq data have been deposited to the GEO database and can be accessed through the accession number GSE228581.

### Coimmunoprecipitation

Coimmunoprecipitation was performed following a previously published protocol (52). Briefly, freshly egressed tachyzoites of the RH iAP2XII-1 strain were harvested, purified by passing through 3 µm polycarbonate membranes, and washed three times with ice-cold PBS. About 10^8^ of parasites were lysed with NP-40 lysis buffer (Beyotime, Shanghai, China) for 30 min on ice and then centrifuged at 13,000 rpm for 10 min. The supernatants of the parasite lysates were first incubated with mouse IgG-conjugated protein A + G magnetic beads (Beyotime, Shanghai, China) at 4°C for 2 h to remove proteins that bind to magnetic beads or mouse IgG non-specifically. Then, the cleared supernatants were added to protein A + G magnetic beads conjugated with mouse anti-HA antibodies (or mouse IgG as control) and incubated overnight at 4°C with rotation. Subsequently, the beads were washed four times with ice-cold TBS, and the bound proteins were subjected to on-bead trypsin digestion and identified by mass spectrometry as described previously (52).

### Statistical analyses

All results were analyzed with Prism 8 (San Diego, CA, USA) except RNA-seq and ChIP-Seq data. Statistical analysis was performed by unpaired two-tailed Student’s *t*-test. All experiments were performed at least three times except ChIP-Seq, which was repeated twice.

## Data Availability

RNA-Seq data generated in this study have been deposited to the GEO database under the accession numbers GSE224934 and GSE228580. ChIP-Seq data have also been deposited to the GEO database under the accession number GSE228581.

## SUPPLEMENTAL MATERIAL

Fig. S1 mbio.01785-23-s0001.pdf TgAP2XII-1 is specifically expressed in tachyzoites.

Fig. S2 mbio.01785-23-s0002.pdf Prolonged depletion of TgAP2XII-1 reduced the growth of parasites.

Fig. S3 mbio.01785-23-s0003.pdf AP2XII-1 depletion changed the division pattern of *Toxoplasma* cells.

Fig. S4 mbio.01785-23-s0004.pdf Quantification of centrosome and apicoplast numbers in AP2XII-1-depleted mutants.

Fig. S5 mbio.01785-23-s0005.pdf Clustering of genes upregulated in the AP2XII-1-depleted mutants.

Fig. S6 mbio.01785-23-s0006.pdf TgAP2XII-1 depletion leads to expression of GRA11b in alkaline-induced bradyzoites.

Fig. S7 mbio.01785-23-s0007.pdf TgAP2XII-1 depletion leads to downregulation of many tachyzoite-specific genes.

Table S1 mbio.01785-23-s0008.xlsx Primers used in this study.

Table S2 mbio.01785-23-s0009.xlsx Gene expression changes in the RH iAP2XII-1 strain after IAA treatment.

Table S3 mbio.01785-23-s0010.xlsx List of genes whose promoters were targeted by AP2XII-1 as revealed by ChIP-seq.

ASM does not own the copyrights to Supplemental Material that may be linked to, or accessed through, an article. The authors have granted ASM a non-exclusive, world-wide license to publish the Supplemental Material files. Please contact the corresponding author directly for reuse.
