# Supplementary material for: AP2XII-1 is a negative regulator of merogony and presexual commitment in Toxoplasma gondii
Source: mBio. 2023 Sep 26;14(5):e01785-23. doi: 10.1128/mbio.01785-23 (PMC10653792; doi:10.1128/mbio.01785-23)
Supplement: Fig. S1 — TgAP2XII-1 is specifically expressed in tachyzoites. [file mbio.01785-23-s0001.pdf]

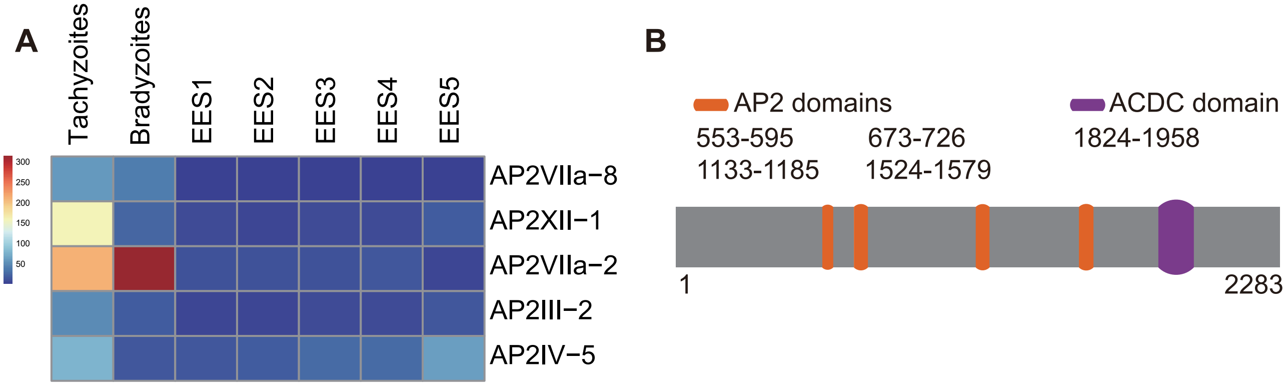

FIG S1 TgAP2XII-1 is specifically expressed in tachyzoites. (A) Expression patterns of five AP2 factors at different life stages of *T. gondii*. Data plotted were from ToxoDB. These five AP2 factors were selected because their mRNA abundance difference between tachyzoites and merozoites were more than 8-fold. (B) Domain structure of TgAP2XII-1.
