# Supplementary material for: AP2XII-1 is a negative regulator of merogony and presexual commitment in Toxoplasma gondii
Source: mBio. 2023 Sep 26;14(5):e01785-23. doi: 10.1128/mbio.01785-23 (PMC10653792; doi:10.1128/mbio.01785-23)
Supplement: Fig. S2 — Prolonged depletion of TgAP2XII-1 reduced the growth of parasites. [file mbio.01785-23-s0002.pdf]

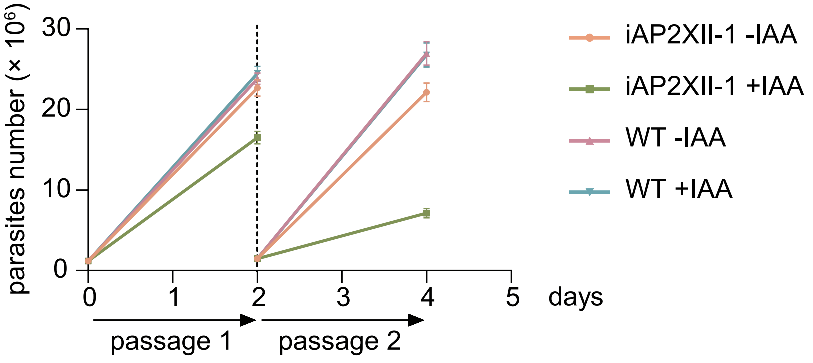

FIG S2 Prolonged depletion of TgAP2XII-1 reduced the growth of parasites. The WT and iAP2XII-1 strains were used to infect HFF cells seeded in T25 flasks (1.2 x10^6 parasites per T25) and cultured with or without IAA for 48 hours (passage 1). Then, the parasites were released from host cells by syringe passage and counted. Subsequently, 1.2 x 10^6 parasites from each condition were passed into fresh HFF monolayers (passage 2), cultured under the same conditions like in passage 1 and parasite growth was monitored by determining the number of parasites after 48 hours of growth.
