# Supplementary material for: AP2XII-1 is a negative regulator of merogony and presexual commitment in Toxoplasma gondii
Source: mBio. 2023 Sep 26;14(5):e01785-23. doi: 10.1128/mbio.01785-23 (PMC10653792; doi:10.1128/mbio.01785-23)
Supplement: Fig. S3 — AP2XII-1 depletion changed the division pattern of Toxoplasma cells. [file mbio.01785-23-s0003.pdf]

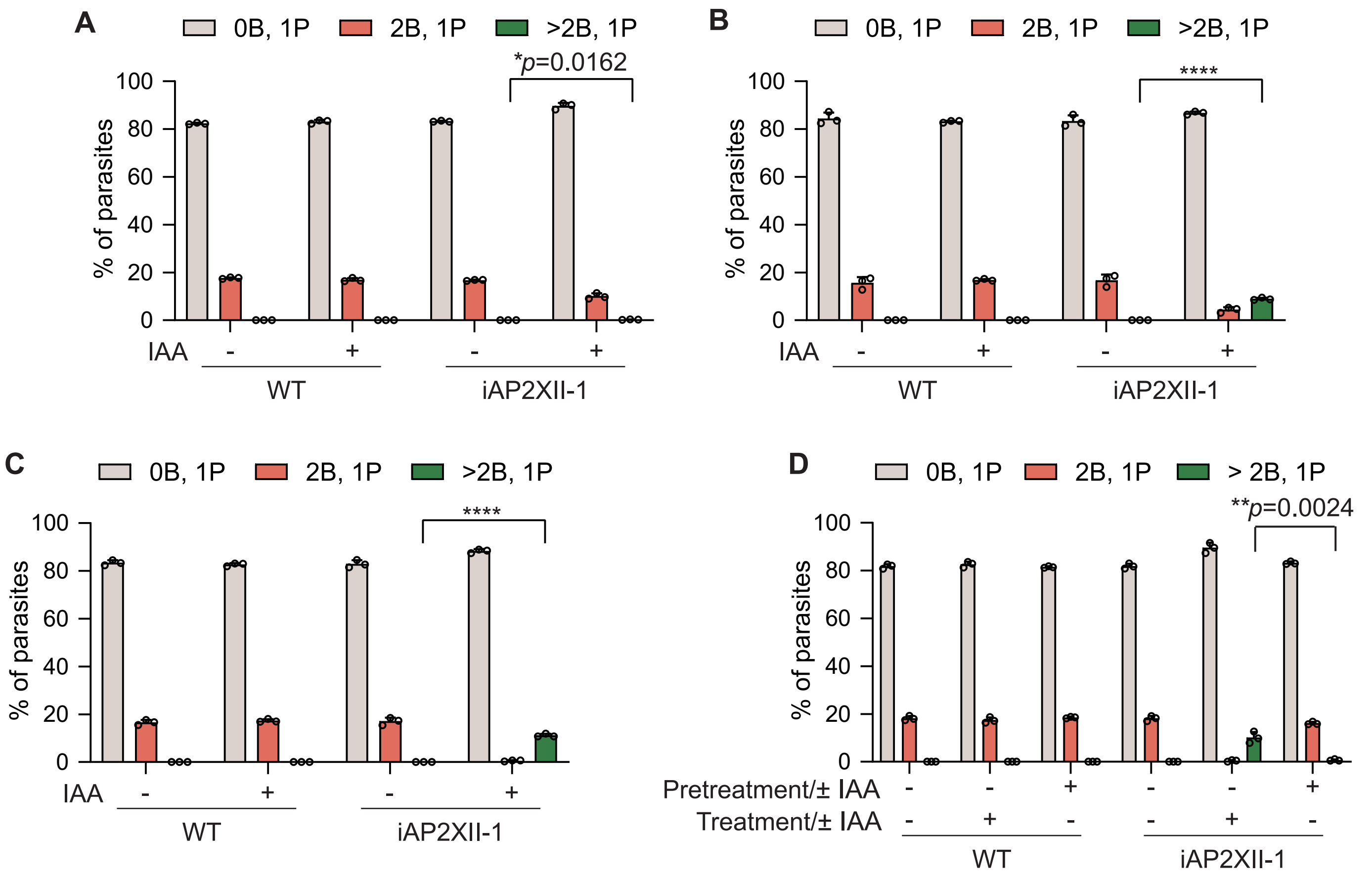

FIG S3 AP2XII-1 depletion changed the division pattern of *Toxoplasma* cells. (A, B and C) The distribution of different budding patterns in indicated strains treated with or without IAA for for 6 h (A), 12 h (B) and 24 h (C) as determined by IFAs. IMC1 and GAP45 were stained to identify the division pattern and the bud number in each parasite. 0B, 1P: no daughter cell budding in the parasite; 2B, 1P: two daughter cells budding in one parasite; >2B, 1P: three or more daughter cells budding in one parasite. More than 300 parasites were analyzed for each biological replicate. Means ± SD of three independent experiments. ****$p < 0.0001$, unpaired two-tailed Student's t-test. (D) Change of cell division pattern caused by AP2XII-1 depletion can be reversed by re-expression of AP2XII-1. Indicated parasites pretreated with or without IAA for 24 h were allowed to infect fresh HFF monolayer and grown for another 24 h under the indicated treatment conditions. Then all samples were subjected to IFA staining to count the bud number in each parasite using IMC1 and GAP45 as markers. More than 450 parasites were analyzed in each biological replicate. Means ± SD of three independent experiments. ****$p < 0.0001$, unpaired two-tailed Student's t-test.
