# Supplementary material for: AP2XII-1 is a negative regulator of merogony and presexual commitment in Toxoplasma gondii
Source: mBio. 2023 Sep 26;14(5):e01785-23. doi: 10.1128/mbio.01785-23 (PMC10653792; doi:10.1128/mbio.01785-23)
Supplement: Fig. S4 — Quantification of centrosome and apicoplast numbers in AP2XII-1-depleted mutants. [file mbio.01785-23-s0004.pdf]

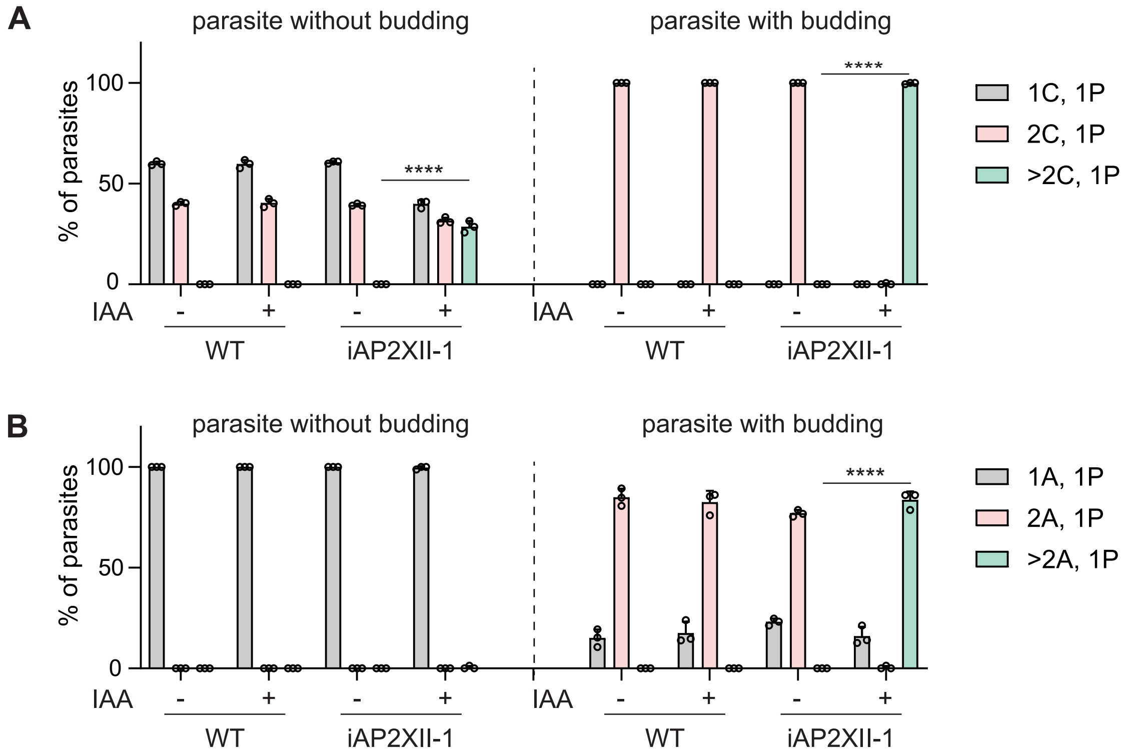

FIG S4 Quantification of centrosome and apicoplast numbers in AP2Ⅻ-1 depleted mutants. (A and B) Quantification of centrosome (A) and apicoplast (B) distribution patterns (1C, 1P: one centrosome in one parasite; 2C, 1P: two centrosomes in one parasite; >2C, 1P: three or more centrosomes in one parasite; 1A, 1P: one apicoplast in one parasite; 2A, 1P: two apicoplasts in one parasite; >2A, 1P: three or more apicoplasts in one parasite) in strains with indicated treatments. The numbers of centrosomes and apicoplasts in parasites were determined by IFA using anti-Centrin1 and anti-PDHe1α, respectively. Parasites with or without daughter cell budding (determined by IMC1 staining) were plotted separately. More than 100 parasites with budding and 300 parasites without budding were analyzed in each biological replicate. Means ± SD of three independent experiments. ****p < 0.0001, unpaired two-tailed Student's t-test.
