# Supplementary material for: AP2XII-1 is a negative regulator of merogony and presexual commitment in Toxoplasma gondii
Source: mBio. 2023 Sep 26;14(5):e01785-23. doi: 10.1128/mbio.01785-23 (PMC10653792; doi:10.1128/mbio.01785-23)
Supplement: Fig. S5 — Clustering of genes upregulated in the AP2XII-1-depleted mutants. [file mbio.01785-23-s0005.pdf]

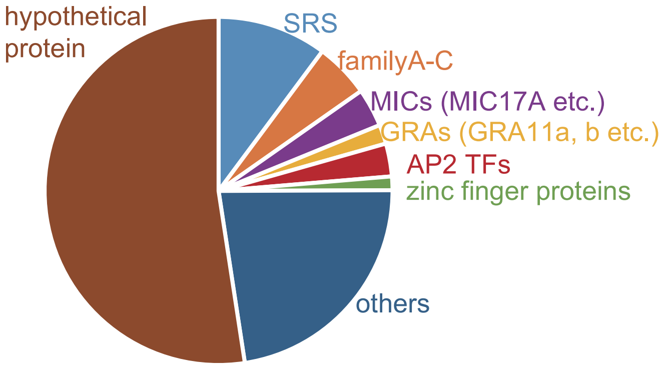

FIG S5 Clustering of genes with increased mRNA abundance (> 2 fold) in the AP2XII-1 depleted mutants, based on their protein sequence features.
