# Supplementary material for: AP2XII-1 is a negative regulator of merogony and presexual commitment in Toxoplasma gondii
Source: mBio. 2023 Sep 26;14(5):e01785-23. doi: 10.1128/mbio.01785-23 (PMC10653792; doi:10.1128/mbio.01785-23)
Supplement: Fig. S6 — TgAP2XII-1 depletion leads to expression of GRA11b in alkaline-induced bradyzoites. [file mbio.01785-23-s0006.pdf]

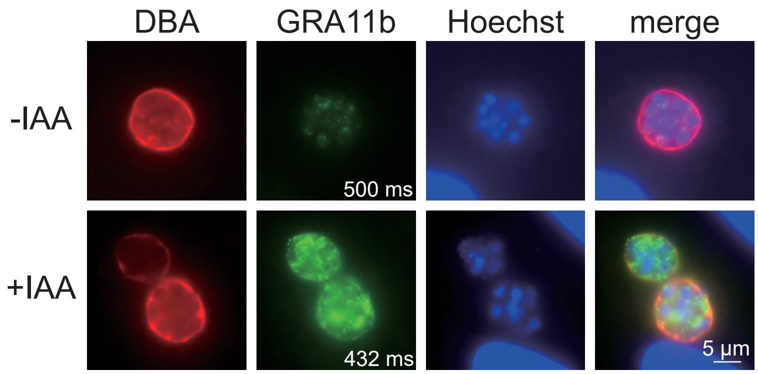

FIG S6 TgAP2XII-1 depletion leads to expression of GRA11b in alkaline induced bradyzoites. The ME49 iAP2XII-1 strain was allowed to grow in the alkaline medium (pH = 8.2) for 3 days to induce bradyzoite transition. Then, the parasites were used to infect fresh HFF monolayers and grown for 48 h in the presence or absence of IAA. Subsequently, samples were fixed and probed with DBA and GRA11b antibodies.
