# Supplementary material for: AP2XII-1 is a negative regulator of merogony and presexual commitment in Toxoplasma gondii
Source: mBio. 2023 Sep 26;14(5):e01785-23. doi: 10.1128/mbio.01785-23 (PMC10653792; doi:10.1128/mbio.01785-23)
Supplement: Fig. S7 — TgAP2XII-1 depletion leads to downregulation of many tachyzoite-specific genes. [file mbio.01785-23-s0007.pdf]

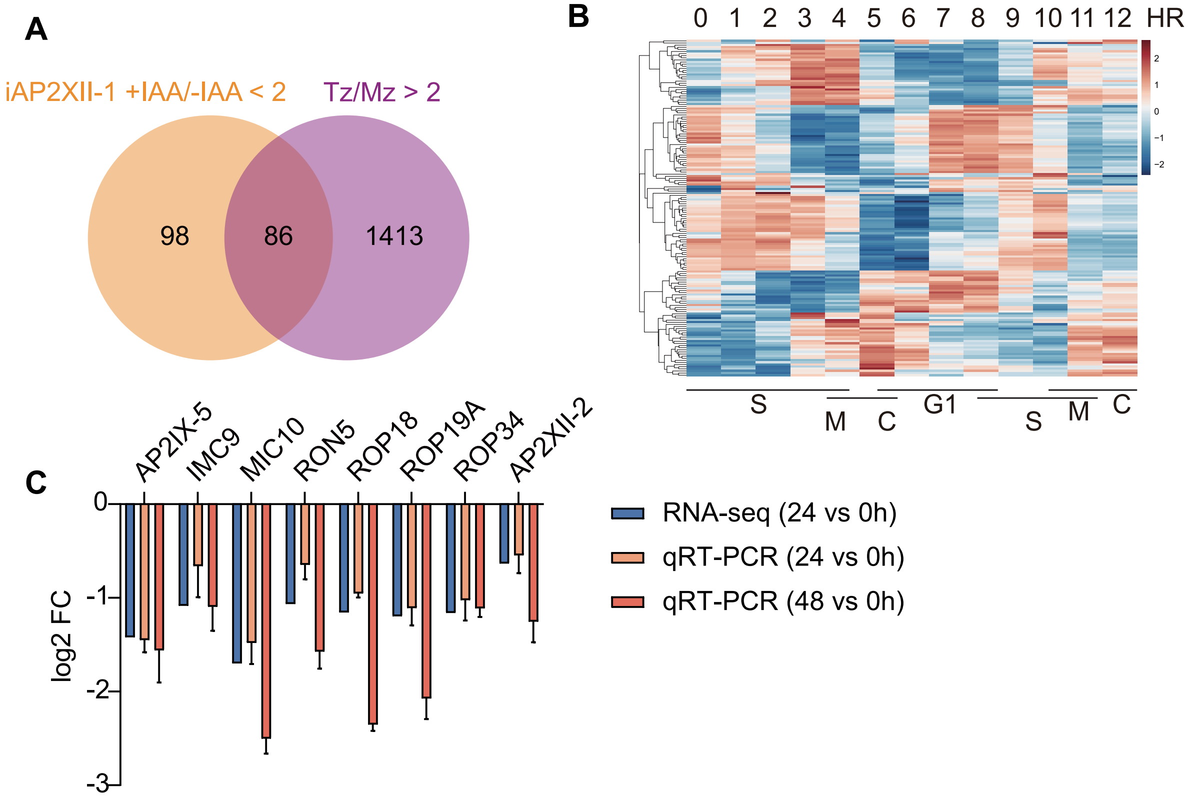


**FIG S7** TgAP2XII-1 depletion leads to reduced transcripts level of many tachyzoite-specific (compared to merozoites) genes. (A) Venn diagram showing the overlap of down regulated genes in AP2XII-1 depleted mutants and tachyzoite specific genes (data from ToxoDB). (B) Heatmap showing the life cycle expression patterns (RMA values) of genes that are down regulated in AP2XII-1 depleted mutants, data plotted were from ToxoDB. (C) Expression changes of selected genes downregulated in AP2XII-1 depleted mutants were verified by qRT-PCR after treating parasites with IAA for 0 h, 24 h or 48 h. Beta tubulin was used as a normalization control.
